# Mental Health Gap Action Programme training in Nigeria: reflections for progressive learning among primary care workers

**DOI:** 10.1192/bji.2021.32

**Published:** 2022-05

**Authors:** Alvina Ali, Nandini Chakraborty

**Affiliations:** 1 Child And Adolescent Psychiatrist, Child And Adolescent Mental Health Unit, Leicestershire Partnership NHS Trust, UK, email: alvina.ali@leicspart.nhs.uk; 2 Consultant Psychiatrist, PEIR Team, Leicestershire Partnership NHS Trust, UK

**Keywords:** Mental health, training, primary care

## Abstract

In the majority of low- and middle-income countries, mental healthcare is delivered by primary care workers. Often, they are the only contact for patients and their families. Although their knowledge base can be limited, they are expected to manage complex cases with few resources. The authors describe their experience of partnership with mental health centres set up by the Nigeria Health Care Project, and training their primary care workers based on the World Health Organization's Mental Health Gap Action Programme. Although the programme was very effective in helping to upskill their knowledge and experience, a need for continued professional development was highlighted. Based on their feedback, multiple evidence-based options are explored, including the use of remote learning and social media (increased significantly around the world because of the COVID-19 pandemic), to help primary care workers improve their knowledge base and maintain their competencies with the limited resources available.

A gap in mental health service provision is recognised as a global health problem. People with mental illness or a learning disability are some of the most stigmatised and abused people in society. In recent years, mental healthcare has shifted increasingly toward early intervention and management in community settings rather than in hospitals. However, many low- and middle-income countries do not have the services or resources to respond to the huge need that exists.

In the majority of countries primary care workers are the first, or in many cases the only, clinical contact available for patients and carers. Studies show that the limited knowledge among health care professionals can be a barrier to providing optimal care in primary care settings.^1^ Often, the treatment provided by them is symptom-based, without an understanding of the diagnostic formulation based on the biopsychosocial model. Hence, clinicians working in rural settings tend to require more diverse skill sets to manage complex conditions with very little resources.^1^

Leicestershire Partnership NHS Trust joined the NHS International Links scheme in 2004, with the aim of improving and strengthening mental health and learning disability care outside the UK and providing opportunities for staff to develop and expand their skills and knowledge. The Nigeria Health Care Project (NHCP) is part of that link.

Nigeria, called the ‘Giant of Africa’, is the seventh most populous country in the world. Up to 20–30% of population suffer from mental disorders.^2^ There are more than 34 000 health facilities, 66% of which were owned by the three tiers of government (federal, state and local government authority). The secondary- and tertiary-level health facilities are mostly found in urban areas, whereas rural areas are predominantly served by primary health care facilities. Problems regarding equity, accessibility, affordability and efficiency of mental health services (which are the overall policy objectives of the revised national health policy of Nigeria) still persist in the country.

## The NHCP

The project was launched in April 1992 to revive the work of the Wesley Guild and medical mission in Nigeria, which began at Ilesha Hospital in 1912.^3^ Beginning with just two (centres) projects in 1992, they are currently involved in funding 16 projects. They provide assessment and treatment for individuals who have become homeless because of mental health problems, and act as rehabilitation centres for residents. The teams are led by a qualified community psychiatric nurse (CPN) and community health workers on each site.^3^

## Mental Health Gap Action Programme

The training provided was based on the World Health Organization's Mental Health Gap Action Programme (mhGAP),^4^ which plays a key role in ‘scaling up services’ across the developing world. It provides competency-based training aimed to build on the existing knowledge and skills of clinicians mainly in a primary care setting.

We delivered mhGAP version 2.0 training to two groups of multidisciplinary clinicians who were part of the NHCP. One group received this training for first the time, and the second group received it as a refresher to revise and reinforce knowledge gained in previously attended training. We used a variety of techniques, such as class-based learning, role-plays and case-based discussions, to help them develop these competencies. Feedback on learning was collected and reflected on at the end of the training, from both groups.

## Feedback and reflection

Both groups were excellent examples of CPN-led projects in the community. There was a high level of interest in learning in both groups. In the post-training feedback, participants reported that it helped them to develop a more reflective mental health practice, becoming more aware of mental health issues, and they were able to integrate new knowledge and skills into their work.

For the group receiving refresher training, it was evident that the topic-based discussions were at a more sophisticated level and seemed to be more management focused. They reported an improved awareness of the need for preventative work at schools and community, especially on addiction and suicide. For the group receiving training for the first time, the focus remained more on improving their assessment skills and general mental health awareness.

There was a difference in the knowledge and skills of different professional groups (CPN and primary care workers). It was clear that formal education at an undergraduate level is not enough to enable primary care workers to continue to manage day-to-day challenges over time, and especially those related to mental health. They both expressed a need for further training as part of their continuous professional development, to allow them to support the rapidly changing mental health needs of the population they covered. There was no existing concept of peer supervision or staff team days to encourage mutual learning.

We explored ideas of peer support and supervision/further training from experienced clinicians (e.g. psychiatrists) in the field. Although both groups were keen to develop the idea of peer support, supervision from experienced psychiatrists and other experts to promote further learning was recognised as a challenge. The main reason was the remote location of their bases and long distance from larger centres where psychiatrists were present, which made it logistically difficult for this option to be implemented. A need to think of more creative accessible solutions was highlighted.

## Discussion

It is known that health promotion and education in the general public and care workers significantly help in healthcare management, especially in developing countries. Primary care workers can play an important and effective role in promoting healthcare, particularly if they are well informed. Previous studies have shown that the attitudes of community volunteers toward people with mental illness improve with educational programmes, which leads to improved treatment and care of individuals who are mentally ill.^5^ The question is how and by whom this can be done effectively, ensuring continuity and sustainability over the long term. Training all staff at once without disrupting clinical services in an already resource-stretched area was impossible. The reason for choosing mhGAP training was also because its framework provides the relevant knowledge and encourages building networks for further supervision.

Psychiatrists can play an important role in upskilling non-medical staff through regular training, teaching and supervision. However, most psychiatrists practicing in low- and middle-income countries are located in university hospitals in urban centres. Majority of them are insulated from the larger community that they serve, and often lack an understanding of patients’ living conditions and needs in rural areas.^6^ CPN-led projects like NHCP have proved to be very effective in bridging that gap. However, there is need for secondary care to link and support primary care clinicians, ensuring their continuous professional development over time. It appears that there are significant challenges in the current processes to link up with the regional- and state-level psychiatric units (as demonstrated in our group setting) for further training and supervision. Also, when support from regional units is offered, it can be *ad hoc* and resource limited.

The other challenges that we found, such as diversity, scope and complexity of primary healthcare and limited infrastructure available to support the population, were very similar to those reported in other projects of this nature. With the COVID-19 pandemic, the availability of such face-to-face learning opportunities seems more distant. However, the pandemic-induced surge in the use of social media has also lead to the use of very innovative ideas across the world, to exchange knowledge and improve our continuous professional development.

There is evidence that many creative ideas have also been tried and implemented successfully even before the pandemic. Some of the following have proven to be very effective and are probably more achievable now, with the use of social media platforms.

### Peer learning

This is cost-effective and improves social connectedness of the participants. It enables them to learn new skills and share good practices, which in turn helps to increase their sense of self-worth and improve their relationships with others.^7^

### Experiential learning

Experiential learning is defined as learning achieved through the appropriate use of current experience. It is one of the educational models that can be used to achieve CPD and improve health service delivery.^8^

### Use of digital media/e-learning

e-Learning is being increasingly used in healthcare education. There is growing evidence that e-learning produces broadly similar outcomes as face-to-face education. However, there is burgeoning interest in how healthcare professionals learn online, and how they transfer their learning into clinical practice. Many countries, like Australia, Nepal and India, have already used digital platforms as an effective way of upskilling clinicians who work in rural settings. The issue is the availability and support in using it appropriately.^9^ There have been trials of using video lectures and on-site skills training successfully, to address the limitations of a conventional training model. The key is that it is accessible and able to transfer the clinical knowledge required.

### Interprofessional programmes

Interprofessional programmes teach and train workers to work together to manage the chronic and complex patient groups effectively. As Church et al indicated, there are many programmes tried and proven to be of benefit to primary care workers, in many countries.^10^ Psychiatry trainees could play a crucial role here, if rotation to rural areas become mandatory part of their training. Support from the government and psychiatrist associations is essential in implementing this. There are obstacles seen in the form of a lack of flexibility in training rotations, isolation from peers and insufficient support when relocating.^11^

There is also a need to develop educational opportunities for trainees placed on these sites, and explore the necessary adaptation of existing curricula to provide optimal learning in rural and remote environments.^12^

All methods described above have a good evidence base, and all have their own advantages and limitations. However, if adapted realistically, they could all be potentially effective.

## Conclusions

Our experience demonstrates that the mhGAP training programme not only improved the knowledge base and skills, but also promoted the social connectedness, of different mental healthcare teams in various settings. As Joynes et al^13^ state, maximising informal learning opportunities and removing barriers to doing so should be a priority for primary care practitioners, managers and educators. The idea for groups to set up peer supervision would certainly help to enhance their mutual support over the long term. We also agree that there is a need for advocacy for them to sustain this support, and accept the fact that there are clear challenges surrounding this. Creative use of social media could be a possible solution, but internet connectivity could still be a problem in such areas. Despite this, there is a huge potential (as demonstrated during the COVID-19 pandemic) for further training and remote supervision through this platform, which is worth future exploration.
